# Ethnopharmacological Significance of *Eclipta alba* (L.) Hassk. (Asteraceae)

**DOI:** 10.1155/2014/385969

**Published:** 2014-10-29

**Authors:** Rownak Jahan, Abdullah Al-Nahain, Snehali Majumder, Mohammed Rahmatullah

**Affiliations:** ^1^Department of Biotechnology & Genetic Engineering, University of Development Alternative, Dhanmondi, Dhaka 1209, Bangladesh; ^2^Department of Pharmacy, University of Development Alternative, Dhanmondi, Dhaka 1209, Bangladesh; ^3^Department of Microbiology and Serology, NH Health, Bangalore 560099, India; ^4^Faculty of Life Sciences, University of Development Alternative, House No. 78, Road No. 11A (new), Dhanmondi, Dhaka 1209, Bangladesh

## Abstract

*Eclipta alba* can be found growing wild in fallow lands of Bangladesh where it is considered as a weed by farmers. Traditional medicinal systems of the Indian subcontinent countries as well as tribal practitioners consider the plant to have diverse medicinal values and use it commonly for treatment of gastrointestinal disorders, respiratory tract disorders (including asthma), fever, hair loss and graying of hair, liver disorders (including jaundice), skin disorders, spleen enlargement, and cuts and wounds. The plant has several phytoconstituents like wedelolactone, eclalbasaponins, ursolic acid, oleanolic acid, luteolin, and apigenin. Pharmacological activities of plant extracts and individual phytoconstituents have revealed anticancer, hepatoprotective, snake venom neutralizing, anti-inflammatory, and antimicrobial properties. Phytoconstituents like wedelolactone and ursolic and oleanolic acids as well as luteolin and apigenin can form the basis of new drugs against cancer, arthritis, gastrointestinal disorders, skin diseases, and liver disorders.

## 1. Background

*Eclipta alba* (L.) Hassk. (also known as* Eclipta prostrata* Roxb.) belongs to the Asteraceae family and is commonly known as false daisy in English and bhringoraj or bhringraj in Bangladesh and India. It is regarded as a weed of ethnomedicinal significance. It is known in the three major forms of traditional medicinal systems in the Indian subcontinent, namely, Ayurveda, Unani, and Siddha, as bhringoraaja, bhangraa, and karissalaankanni, respectively. The Ayurvedic Pharmacopoeia of India considers the plant as hepatoprotective [1]. The full taxonomic hierarchy is shown below in Table 1.

Considering the ethnomedicinal significance of the plant, it was of interest to review the ethnopharmacological reports on the plant and selective phytoconstituents through data base searches (PubMed, Scopus, Google Scholar).

## 2. Reported Phytochemical Constituents

**Figure 1:**
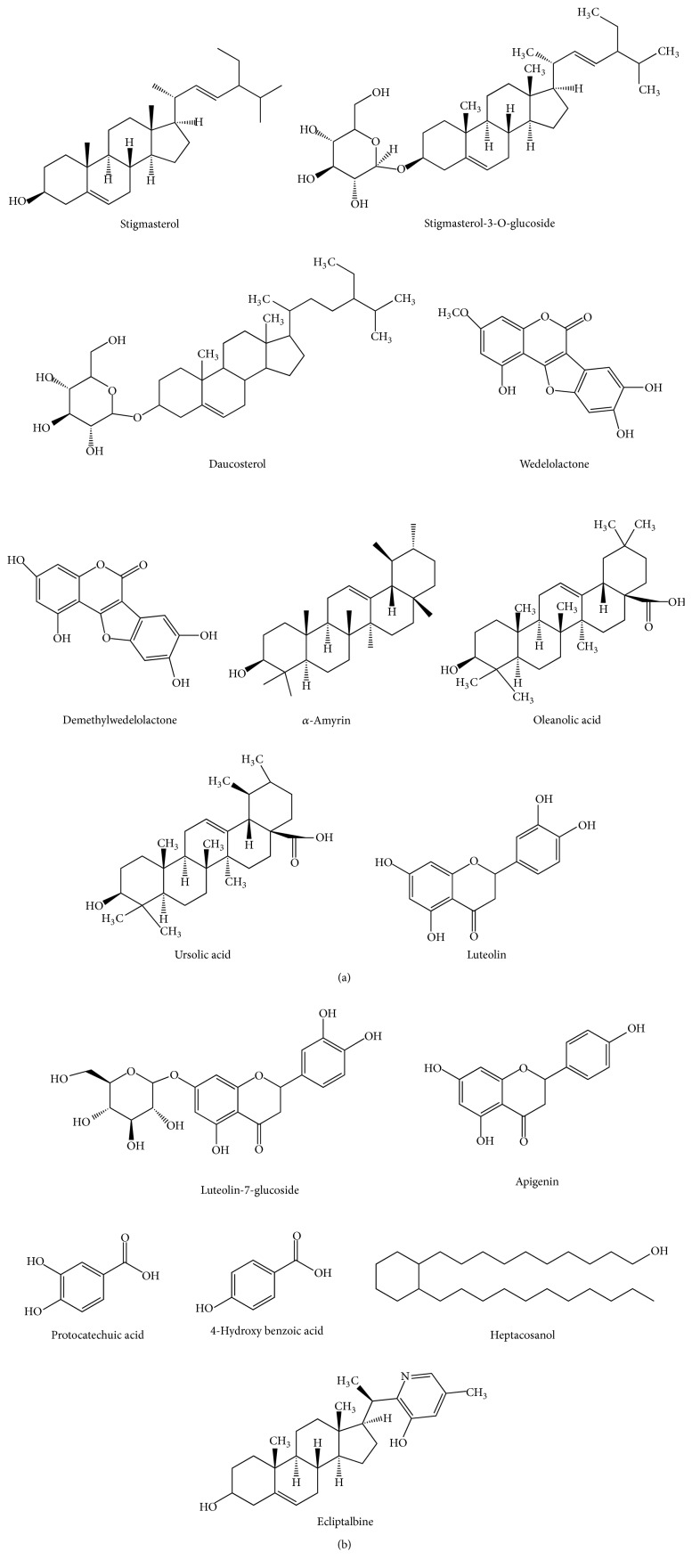
Structures of several phytoconstituents of* E. alba*.

The plant reportedly contains a number of bioactive chemicals [2]. The reported constituents are shown in Table 2. The structures of several phytoconstituents are shown in Figure 1.

## 3. Ethnomedicinal Reports

The plant and plant parts are used fortreatment of a variety of diseasesby folk medicinal practitioners and tribal medicinal practitioners of the Indian subcontinent. Ethnomedicinal uses of the plant have been reported from Bangladesh, India, Nepal, and Pakistan (Table 3).

The available ethnomedicinal reports indicate that although there are a variety of diseases treated with the plant or plant parts, the major uses are limited to treatment of gastrointestinal disorders, respiratory tract disorders (including asthma), fever, hair loss and graying of hair, liver disorders (including jaundice), skin disorders, spleen enlargement, and cuts and wounds.

## 4. Pharmacological Activity Reports

### 4.1. Hepatoprotective Activity

The protective effect of the plant against carbon tetrachloride-induced acute liver damage has been reported [3]. Coumestans (wedelolactone and demethylwedelolactone) have been mentioned as the possible components behind the protective effect on liver as well as against liver disorders; both compounds exhibited antihepatotoxic activity in assays employing CCl_4_—(carbon tetrachloride), GalN—(galactosamine), and phalloidin-cytotoxicity in rat hepatocytes. They also showed a significant stimulatory effect on liver cell regeneration [4]. Alcoholic extract of* E. alba* was found to have good antihepatotoxic activity as assessed in CCl_4_-induced liver damage in albino rats through liver to body weight ratio, pentobarbitone sleep time, serum levels of glutamate pyruvate transaminase (GPT) and glutamic oxaloacetic transaminase (GOT), alkaline phosphatase (ALP), and bilirubin. In CCl_4_-administered rats, there was an increase in liver weight, pentobarbitone sleep time, and elevated GOT, GPT, SALP, and serum bilirubin levels. The alcoholic extract at a dose of 200 mg/kg significantly reversed these effects [5].

The hepatoprotective effect of ethanol/water (1 : 1) extract (Ea) of the plant has been studied in CCl_4_-induced hepatotoxicity in rats. Ea significantly counteracted CCl_4_-induced inhibition of the hepatic microsomal drug metabolising enzyme amidopyrine N-demethylase and membrane bound glucose 6-phosphatase but failed to reverse the very high degree of inhibition of another drug metabolising enzyme aniline hydroxylase. The loss of hepatic lysosomal acid phosphatase and alkaline phosphatase by CCl_4_ was significantly restored by Ea. It was suggested that the hepatoprotective effect of Ea may be due to its regulating of the levels of hepatic microsomal drug metabolising enzymes [43].

The ethanolic extract of leaves of the plant has been fractionated into three parts (hot water insoluble (EaI), ethyl acetate fraction of hot water soluble (EaII), and remaining hot water soluble fraction (EaIII)) and each fraction studied for hepatoprotective activity against CCl_4_-induced hepatotoxicity in rats and mice. Hepatoprotective activity was determined on the basis of their effects on parameters like hexobarbitone sleep time, zoxazolamine paralysis time, bromsulfalein clearance, serum transaminases (GPT, GOT), and serum bilirubin. All the experimental parameters were increased by CCl_4_; fraction EaII (10–80 mg/kg, p.o.) dose-dependently and significantly reversed these increases. Fraction EaII was found to contain coumestan wedelolactone and demethylwedelolactone as major components with apigenin, luteolin, 4-hydroxybenzoic acid, and protocatechuic acid as minor constituents [44].

Hepatitis C virus (HCV) inhibitory activity has been reported for* E. alba* extract. Phytochemical analysis of the extract revealed the presence of three compounds, namely, wedelolactone, luteolin, and apigenin. These compounds exhibited dose-dependent inhibition of HCV replicase* in vitro*, and anti-HCV replication activity in the cell culture system. The results suggest that the plant or individual components have the potential to be used against HCV [45].

Ethanol extract of whole plant was tested for hepatoprotective effect against paracetamol-induced hepatotoxicity in mice. Treatment with 100 and 250 mg of the extract per 100 kg body weight showed significant reductions in paracetamol-induced serum alanine aminotransferase (ALT, also known as GOT) levels. At the same time, histopathological studies showed marked reductions in paracetamol-induced fatty degeneration and centrizonal necrosis in liver of extract-treated mice [46].

An alcoholic extract of freshly collected* Eclipta alba* exhibited dose-dependent (62.5–500 mg/kg p.o.) significant hepatoprotective activity against carbon tetrachloride-induced liver injury in rats and mice as determined through various tests like hexobarbitone-induced sleep, zoxazolamine-induced paralysis, bromsulfalein (BSP) clearance, serum levels of transaminases, bilirubin, and protein [47].

A combination of ethanolic extract of* E. alba* leaves and* P. longum* seeds demonstrated better hepatoprotective action against CCl_4_-induced hepatotoxicity in rats than either extract alone. Serum marker enzymes like alanine aminotransferase (ALT/GOT), aspartate aminotransferase (AST, also known as GOT), acid phosphatase (AP), lactate dehydrogenase (LDH), *γ*-glutamyl transferase (GGT), and 5′-nucleotidase were elevated with carbon tetrachloride treatment, which were restored towards normalization by the combined extract. At the same time, changes in biochemical parameters like total protein, total bilirubin, total cholesterol, triglycerides, and urea were restored to near normal levels with the combined extract [48].

Administration of fresh leaf powder (500 mg/kg) to rats was observed to lead to significant hepatoprotective action in paracetamol-induced liver toxicity in rats. Histopathological studies indicated that paracetamol-administered rat liver showed severe congestions, hydropic degeneration, and occasional necrosis, while leaf-administered rat liver showed decreased hepatocyte damage. At the same time, paracetamol-induced elevated levels of serum ALT, AST, alkaline phosphatase (ALP), LDH, and GGT as well as paracetamol-induced changes in serum proteins, bilirubin, cholesterol, and triglycerides were restored to normal levels with the leaf powder [49].

In CCl_4_-induced hepatotoxicity in rats, methanol extract of leaves and chloroform extract of roots of* E. alba* showed significant reductions of lysosomal enzymes in serum from the elevated levels induced by carbon tetrachloride. At the same time CCl_4_-induced elevated serum GOT, GPT, ALP, and bilirubin levels were also restored towards normalization with administration of both extracts [50].

The hepatoprotective activity of ethanol extract of* E. alba* whole plants was studied in rats given ethanol for 21 days. Ethanol administration led to hepatic damage as manifested by histopathological changes, increase in thiobarbituric acid-reactive substances (TBARS), decrease in reduced glutathione (GSH), superoxide dismutase (SOD), and catalase (CAT), and increase in glutathione peroxidase (GPx) in liver. Histopathological changes indicated that, in alcohol-treated animals, liver showed hepatocytic necrosis and inflammation in the centrilobular region with portal triaditis. These toxic effects were reversed with coadministration of extract with ethanol [51].

Aqueous extract of leaves of the plant has been found to offer hepatoprotectivity against paracetamol-induced liver damage. Paracetamol-induced increases in TBARS were reduced by the aqueous extract, and paracetamol-induced decreases in GSH were also reversed by the extract. Catalase was also decreased in paracetamol-treated groups, which was also reversed by coadministration of the extract [52].

The alcoholic and aqueous extract of* E. alba* leaves was tested for hepatoprotective activity against paracetamol-induced liver damage in albino rats. The alcoholic extract demonstrated significant hepatoprotective effects. The alcoholic extract-treated rats of group III revealed marked hepatoprotection as there was significant (*P* < 0.01) reduction in SGOT, SGPT, ALP, total bilirubin, and direct bilirubin and a significant (*P* < 0.01) increase in total protein and albumin as compared to paracetamol treated group [53].

The aqueous leaf extract (85%) of* E. alba* was examined for hepatoprotective effects in CCl_4_-induced hepatotoxicity in male albino rats. CCl_4_ induced oxidative stress in rats resulting in oxidative injury as manifested by increases in TBARS and hydroperoxides and augmented levels of serum AST, ALT, and ALP. At the same time there were depleted levels of SOD, CAT, GPx, and glutathione-S-transferase (GST). The aqueous extract given at a dose of 250 mg per kg body weight reversed these changes and brought them back to normal levels. The results suggest that increases in oxidative stress play a vital role in development of hepatic injury, which can be ameliorated through administration of aqueous extract of the leaves [54].

A polyherbal formulation (Ayush-Liv.04) containing* E. alba* (along with* Clitoria ternatea, Asparagus racemosus, Alpinia galanga*, and milk tuttam, i.e., copper containing stone) showed hepatoprotective activity against CCl_4_ and ethanol induced liver damage in rats. Elevated levels of serum AST, ALT, ALP, acid phosphatase, and bilirubin were significantly lowered in the polyherbal formulation-administered rats [55].

The ethanolic extract of a polyherbal formulation containing leaves of* Melia azadirachta*, seeds of* Piper longum*, and whole plants of* E. alba* has been evaluated for hepatoprotective effects against CCl_4_-induced hepatic damage in male albino rats. The substantially reduced levels of SOD, CAT, GPx, GST, and glutathione reductase (GR) due to CCl_4_ were restored to normal with the extract [56].

Hepatoprotective effects of ethanolic extract of* E. alba* leaves and leaf callus were examined in a model of CCl_4_-induced acute hepatotoxicity in albino rats. Liver damage was assessed by measuring serum parameters like GOT, GPT, ALP, albumin, and total protein, as well as histopathological examination. Oral administration of the extract at 250 and 300 mg/kg, respectively, showed hepatoprotective effects as demonstrated by restoring to near normal levels the serum parameters and improving hepatic lesions caused by carbon tetrachloride [57].

### 4.2. Hair Growth Promoting Activity

Petroleum ether and ethanol extract of* E. alba* has been tested in albino rats for promoting hair growth activity. The extracts were incorporated into oleaginous cream (water in oil cream base) and applied topically on shaved denuded skin of male albino rats. The extracts significantly reduced hair growth time by half, as compared to nontreated control animals. Quantitative analysis of hair growth after treatment with petroleum ether extract (5%) exhibited greater number of hair follicles in anagenic phase (69 ± 4) which were higher as compared to control (47 ± 13) [58].

The methanol extract of the plant has also been tested for its efficacy for promoting hair growth in pigmented C57/BL6 mice, preselected for their telogen phase of hair growth. In these species, the truncal epidermis lacks melanin-producing melanocytes and melanin production is strictly coupled to anagen phase of hair growth. Telogen to anagen transition was assessed following topical administration of the extract. A dose-dependent transition of telogen to anagen phase of hair growth was observed following extract treatment; with an extract dose of 3.2 mg/15 cm^2^, 87.5% animals showed anagen phase of growth, while with an extract dose of 1.6 mg/15 cm^2^, 50% of the animals showed the transition from telogen to anagen phase [59].

A polyherbal formulation containing* E. alba*,* Hibiscus rosa-sinensis*, and* Nardostachys jatamansi* exhibited excellent hair growth activity in Wistar albino rats. Hair growth initiation time and time required for complete hair growth were significantly reduced. Treatment with the formulation resulted in greater number of hair follicles in the anagenic phase [60].

### 4.3. Antidiabetic Activity

The beneficial effects of the plant in diabetes have been reported. An Ayurvedic formulation consisting of* Withania somnifera, Tinospora cordifolia, Eclipta alba, Ocimum sanctum, Picrorhiza kurroa* and shilajit, at doses of 100 and 200 mg/kg, p.o. administered once daily for 28 days to streptozotocin- (STZ-) induced diabetic male CF strain rats, induced a dose-related decrease in STZ hyperglycemia and attenuation of STZ induced decrease in pancreatic islet superoxide dismutase (SOD) activity. It has been suggested that the STZ-induced hyperglycemia was the consequence of decreased islet SOD in islets [61].

In alloxan-diabetic rats, oral administration of leaf suspension of* E. alba* (2 and 4 g/kg body weight) for 60 days resulted in significant reduction in blood glucose (from 372.0 ± 33.2 to 117.0 ± 22.8), glycosylated hemoglobin HbA(1)c, a decrease in the activities of glucose-6 phosphatase and fructose 1,6-bisphosphatase, and an increase in the activity of liver hexokinase, all of these activities being beneficial for amelioration of hyperglycemia and other diabetes-related complications [62].

The antidiabetic effect of* E. alba* ethanolic extract has been investigated for possible beneficial effects against hyperglycemia and diabetic nephropathy in STZ-diabetic rats. Single dose treatment of the extract was found to significantly lower blood glucose level by 17.6% after 5 h of oral administration at a dose of 250 mg/kg. Treatment of STZ-diabetic animals for 10 weeks with the above dose level significantly reduced the elevated levels of blood glucose, %HbA1C, urea, uric acid, and creatinine and significantly increased the depressed serum insulin level. The extract exerted a significant inhibitory effect on *α*-glucosidase in a noncompetitive manner with an IC_50_ value of around 54 *μ*g per mL and was found inhibitory to eye lens aldose reductase with an IC_50_ value of about 4.5 *μ*g per mL. Inhibition of *α*-glucosidase and aldose reductase was postulated to be the reason behind the other observed effects [63]. A bioactivity-guided isolation approach based on *α*-glucosidase inhibition led to the isolation of four echinocystic acid glycosides of which eclalbasaponin VI was found to be the most potent (IC_50_ 54.2 ± 1.3 microM) [64].

### 4.4. Analgesic and Anti-Inflammatory Activities

Analgesic activity of alcoholic extract of* E. alba* has been determined through tail flick, hot plate, and writhing methods in rats and mice. In all three methods, the extract at a dose of 200 mg/kg demonstrated significant analgesic and antinociceptive effects [65].

Hydroalcoholic extract of the plant showed significant antinociceptive activity in acetic acid-induced writhing tests in rodent model at a dose of 200 mg/kg p.o. The extract further showed analgesic effects in formalin tests, with the inhibition occurring in the second phase of the response [66].

The analgesic activity of ethanol extract of* E. alba* whole plants as well as a total alkaloid fraction was seen in experiments with albino mice by using standard experimental models such as the tail clip method, the tail flick method, and the acetic acid induced writhing response. The results from this study showed that both the ethanol extract and the total alkaloids produced good analgesic activity in all the different models of analgesia tested. Total alkaloid fraction showed better analgesic activity than ethanolic extract [67].

The anti-inflammatory effect of the plant was evaluated using carrageenan, mediators such as histamine and serotonin induced paw oedema, and cotton pellet induced granuloma tests for their effect on acute and chronic phase inflammation models in rats. The results indicated potent anti-inflammatory activity of the plant in all the models tested [68]. Cumulatively, the reports suggest that the plant can prove valuable as both a central and peripheral analgesic agent.

### 4.5. Skin Diseases

Leaves of* E. alba* are used to get rid of ectoparasites in dogs in Trinidad and Tobago [69]. An Ayurvedic formulation containing* E. alba* powder has been shown to provide complete remission to 22.6% and checked the recurrence of the disease in 89.5% patients of “Vicharchika” (eczema) [70].

The antioxidant and protective effect of water extract of* E. alba* against ultraviolet- (UV-) irradiation-induced damage has been investigated. The extract had a potent effect in scavenging 2,2-diphenyl-1-picrylhydrazyl (DPPH), superoxide radicals, and chelating ferrous ion, exhibiting IC_50_ values, respectively, of 0.23 mg/mL, 0.48 mg/mL, and 1.25 mg/mL. The total phenol content of the extract was 176.45 mg gallic acid equivalents. The extract was also seen to absorb UVA and UVB irradiation and demonstrated a dose-dependent protection of HaCaT human keratinocytes and mouse fibroblasts 3T3 cells against UVB-induced cytotoxicity. The protective effect against skin cell damage was attributed to a synergistic effect between chlorogenic acid and other active components present in the extract [71].

### 4.6. Neuropharmacological Activities

The aqueous and hydroalcoholic extracts of* E. alba* have been evaluated for sedative, muscle relaxant, anxiolytic, nootropic, and antistress activities at doses of 150 and 300 mg/kg, p.o. The findings indicated nootropic activity of the aqueous extract (300 mg/kg, p.o.) and its hydrolyzed fraction (30 mg/kg, p.o.). The aqueous extract and the hydrolyzed fraction were observed to provide protection against cold restraint induced gastric ulcer formation and also normalized the white blood cell count in the milk induced leukocytosis challenge model [72].

The aqueous extract of leaves of* E. alba* has been examined for its memory enhancing quality. Doses of 100 and 200 mg of extract suspension in water (per kg body weight) were administered to rats to evaluate transfer latency (TL) on an elevated plus maze. This method gives a measure of acquisition and retrieval learning. Spatial habitual learning tests were conducted with mice at the aforementioned two doses. In this method, mice were placed at the center of open-field apparatus to assess spatial habitual learning, observed for 20 minutes for rearing and time spent during rearing for 30 minutes, 24 hours and 96 hours and 144 hours. The extract at both doses produced a significant decrease in TL in rats and the amount of rearing in mice. The results indicate an extract-induced improvement in cognitive functions, which was attributed to the presence of luteolins in the extract [73].

Aqueous extract of* E. alba* has been tested for its ability to reduce aggression through foot shock-induced aggression and water competition tests. Minimization of aggression in both tests was observed with the extract at doses of 100 and 200 mg/kg [74].

Methanolic extract of* E. alba* whole plant has been shown to ameliorate oxidative stress-induced mitochondrial dysfunction in an animal (rat) model of Alzheimer's disease (evaluation of short-term memory using elevated plus maze model). Mitochondrial function was determined through MTT [3-(4,5-dimethylthiazol-2-yl)-2,5-diphenyltetrazolium bromide] assay. Synaptosomal fractions of scopolamine hydrobromide-treated rats exhibited a significant decrease in MTT reduction, which was prevented by the extract at a dose of 200 mg/kg. Scopolamine significantly increased transfer latency in rats indicative of amnesia or impairment of memory. Transfer latency of rats using the elevated plus maze model was also dose-dependently decreased (reversal of scopolamine-induced increase) by the extract thus demonstrating increase in memory [75]. The extract showed high phenolic and flavonoid contents, which might have contributed to amelioration of oxidative stress.

### 4.7. Antioxidant Activity

The methanol and hydrolyzed extract of* E. alba *has been assessed for its antioxidant potential in both* in vitro* and* ex vivo* models. The* in vitro* antioxidant activity was evaluated through 2,2-diphenyl-1-picrylhydrazyl (DPPH) free radical scavenging and nitric oxide radical inhibition activity. The* ex vivo* antioxidant activity was determined through lipid peroxidation inhibitory activity on mice liver homogenate by thiobarbituric acid-reactive substances (TBARS) method. The methanolic extract and hydrolyzed extract both showed potent antioxidant activity in both models in proving to be powerful scavengers of DPPH free radicals and nitric oxide radicals, as well as being inhibitors of lipid peroxidation [76]. Antioxidant activity as assessed by DPPH free radical scavenging methods has also been described for ethanol extract of the plant [77].

Methanolic and aqueous extracts of* E. alba* demonstrated antioxidant activity in hydrogen peroxide scavenging assays, total antioxidant capacity, and through reducing ability assay [78]. The antioxidant potential of the plant methanolic extract has been shown through DPPH free radical scavenging and 2,2′-azinobis-(3-ethylbenzthiazoline-6-sulfonic acid) (ABTS) assays [79]. The ethanolic extract of the plant also demonstrated antioxidant potential in DPPH and ABTS assays [80]. Ethanol and ethyl acetate extracts of leaves of the plant showed antioxidant activity in the ferric thiocyanate method; aqueous and hexane extracts also showed antioxidant effects but less than ethanol and ethyl acetate extracts [81].

The possible cerebroprotective and antioxidant effect of hydroalcoholic extract of* E. alba* has been evaluated in global cerebral ischemia in rats. The global cerebral ischemia-reperfusion injury was induced by occluding bilateral common carotid arteries (BCCA) for 30 min, followed by 4 h reperfusion. BCCA caused significant depletion in superoxide dismutase (SOD), glutathione peroxidase (GPx), reduced glutathione (GSH), catalase (CAT), glutathione-S-transferase (GST), and glutathione reductase (GR) and significant increase in malondialdehyde (MDA) in brain. Pretreatment with hydroalcoholic extract significantly reversed the levels of biochemical parameters and significantly reduced the edema and cerebral infarct size as compared to the ischemic control group [82].

### 4.8. Antimicrobial Activity

Various solvent (petroleum ether, benzene, chloroform, acetone, methanol, and aqueous) extracts of* E. alba* were found to be active against clinical isolates from oral cancer cases. These isolates included various bacteria like* Staphylococcus aureus, Escherichia coli, Staphylococcus epidermis, Pseudomonas aeruginosa, Klebsiella pneumoniae, Proteus mirabilis*, and* Proteus vulgaris* and funguses like* Candida albicans* and* Aspergillus fumigatus* [83].

Ethanol and ethyl acetate extracts of leaves of the plant have been found to be active against* E. coli, K. pneumoniae, Shigella dysenteriae, Salmonella typhi, P. aeruginosa, Bacillus subtilis*, and* S. aureus* with Minimum Inhibitory Concentrations (MIC) ranging from 4.5 to 90 *μ*L/mL [81].

Hexane extract of aerial parts of the plant reportedly showed antibacterial activity against* S. aureus, Bacillus cereus, E. coli, S. typhi, K. pneumoniae, Streptococcus pyogenes*, and* P. aeruginosa*, whereas acetone, ethanol, methanol, and aqueous extracts showed intermediate activity against* S. aureus, B. cereus, E. coli, S. typhi, K. pneumoniae, P. aeruginosa, P. mirabilis*, and* S. pyogenes* [84]. The aqueous extract showed good activity against* S. pyogenes, B. cereus, E. coli*, and* P. aeruginosa*.

The susceptibility of various extracts of* E. alba* was tested against nine different test organisms using the well diffusion method. The n-butanol extract showed inhibitory activity against all nine species, namely,* B. cereus*,* B. subtilis, C. albicans, Erwinia carotovora, E. coli, K. pneumoniae, P. aeruginosa, S. typhi*, and* S. aureus*. Petroleum ether, dichloromethane, methanol and aqueous extracts showed varying levels of inhibition against some of these microorganisms [85].

Aqueous extract of leaves, stems, and flowers of* E. alba* has been screened for inhibitory activity against multiple test organisms. The leaf extract was effective against* Enterobacter cloacae* and* K. pneumoniae*; stem extract was effective against* E. cloacae, Enterococcus faecalis, K. pneumoniae*, and* Staphylococcus saprophyticus*, while flower extract was effective against* P. vulgaris, S. aureus*, and* S. saprophyticus* [86]. Aqueous and ethanolic extracts of leaves also reportedly showed moderate inhibitory activities against* S. aureus, E. coli, P. vulgaris, P. aeruginosa, C. albicans*, and* Aspergillus niger*, when tested by agar plate disc diffusion method [87].

Antimicrobial activity of petroleum ether, ethyl acetate, ethanol and aqueous extract of* E. alba* were screened for inhibitory activity by agar well diffusion method against* B. subtilis, S. aureus, P. mirabilis, B. cereus, E. coli, Salmonella enterica* serv.* typhi, P. aeruginosa, S. epidermis*, and* C. albicans*. Maximum numbers of the organisms tested were inhibited by the ethyl acetate fraction with zones of inhibition ranging from 11 ± 1 mm to 22 ± 1 mm, with maximum activity against* B. cereus*. The ethanol extract demonstrated maximum inhibitory activity against* E. coli*. Petroleum ether extract inhibited only* B. cereus*, while aqueous extract was found to inhibit* B. subtilis, B. cereus, S. aureus*, and* C. albicans* [88].

The antimicrobial activity of methanol, acetone, and aqueous extracts of leaves of three morphotypes of* E. alba* has been examined by the disc diffusion method against the Gram positive bacteria—*S. aureus* and* B. subtilis*—and Gram negative bacteria—*K. pneumoniae, E. coli*, and* P. aeruginosa*. The extracts demonstrated inhibitory activity against all bacteria against* P. aeruginosa*. The aqueous extract was found to be the least effective. The acetone extract showed more antimicrobial activity against* S. aureus, E. coli*, and* K. pneumoniae* than methanol extract. The methanol extract showed maximum activity against* B. subtilis* [89].

An aqueous extract of leaves of* E. alba* has been shown to inhibit the fungus* Fusarium oxysporum* as determined by the agar plate disc diffusion method [90]. Methanolic extract of aerial parts of the plant showed maximum inhibitory activity against* S. epidermis, S. aureus*, and* Salmonella typhimurium*. Wedelolactone, isolated from the ethyl acetate fraction of aerial parts, showed enhanced antimicrobial activity and as such can be the responsible agent behind the observed antimicrobial effects [91]. Eclalbasaponin, another phytochemical constituent of the plant, has been shown to be responsible for the inhibitory activity of the plant against* B. subtilis* and* P. aeruginosa* [92]. This inhibitory activity has been attributed to disruption of bacterial cell membrane leading to loss of bacterial cell viability.

### 4.9. Antimalarial Activity

The antimalarial activity of leaf extract of* E. alba* has been tested against* Plasmodium berghei* ANKA strain in mice. The methanolic leaf extract (250–750 mg/kg) produced a dose-dependent chemosuppression or schizontocidal effect during early and established infection and high mean survival time (m.s.t.) values particularly in the group administered 750 mg/kg/day of extract. The plant extract also exhibited repository activity [93].

Mosquito larvicidal and ovicidal activities of crude hexane, ethyl acetate, benzene, chloroform, and methanol extracts of the leaves of* E. alba* were tested against the early third-instar larvae of* Anopheles stephensi* (Liston) (Diptera: Culicidae). Larval mortality was observed 24 hours following extract exposure. The highest larval mortality was observed with methanol extract (LC_50_ = 112.56 ppm, LC_90_ = 220.68 ppm). Percent hatchability was also found to be inversely proportional to concentration of the extract. Mortality of 100% was exerted with methanol extract at 200 ppm. Thus this plant could prove to be useful in combating malaria through its larvicidal and ovicidal activities [94].

Larvicidal and ovicidal activities of benzene, hexane, ethyl acetate, methanol and chloroform leaf extract of* E. alba* against dengue vector,* Aedes aegypti*, has also been examined. Maximum larvicidal activity was observed with the methanol extract. The LC_50_ values of benzene, hexane, ethyl acetate, methanol and chloroform extract of* E. alba* against early third- instar larvae of* A. aegypti* were 151.38, 165.10, 154.88, 127.64, and 146.28 ppm, respectively. The methanol extract was found to be most effective for ovicidal activity against* A. aegypti*. The methanol extract exerted 100% mortality (zero hatchability) at 300 ppm [95].

The adulticidal and repellent activities of crude hexane, ethyl acetate, benzene, chloroform, and methanol extracts of leaf of* E. alba* were assayed for their toxicity against two important vector mosquitoes, namely,* Culex quinquefasciatus* and* Aedes aegypti* (Diptera: Culicidae).* C. quinquefasciatus* is the vector of* Wuchereria bancrofti*, avian malaria, and arboviruses including St. Louis encephalitis virus, Western equine encephalitis virus, and West Nile virus. All extracts showed moderate adulticide effects. The extracts also had concentration-dependent mosquito repellent activities [96]. The methanol extract of leaves of* E. alba* also reportedly demonstrated maximum adulticidal as well as repellent activities against* A. stephensi* compared to benzene, hexane, ethyl acetate, and chloroform extracts [97].

### 4.10. Cardiovascular Effects

The effect of administration of dried* E. alba* leaf powder (3 g per day) has been studied in mild hypertensive subjects. Subjects were given six capsules (500 mg powder per capsule) in three doses per day for 60 days. When compared with placebo given control groups, the results showed that* Eclipta*-supplemented group showed a marked reduction in mean arterial pressure by 15%, total cholesterol (17%), low-density lipoprotein fraction (24%), triglycerides (14%), very-low-density lipoprotein fraction (14%), and plasma lipid peroxides (18%). There was a marked increase in urine volume (34%), urine sodium (24%), serum vitamin C (17%), and serum tocopherols (23%) in the* Eclipta*-administered group. The findings indicated that leaf powder possessed diuretic, hypotensive, and hypocholesterolemic properties and helps in the alleviation of oxidative stress-induced complications in hypertensives [98].

Ethanolic extract of leaves and leaf calluses of* E. alba* was examined for cardiac inhibitory activity in isolated frog hearts. The extracts showed negative ionotropic and negative chronotropic effects as well as reduction in cardiac output. Callus extract demonstrated a higher cardiac inhibitory effect than leaf extract at 20 mg doses. The callus extract was also found to antagonize the effects of adrenaline [57].

### 4.11. Immunomodulatory Effects

The immunostimulatory effects of feeding aqueous extract of leaves of* E. alba* have been studied in tilapia fish (*Oreochromis mossambicus*). Fish were fed diet containing extract at 0, 0.01. 0.1, and 1% levels of diet. After each week, nonspecific humoral (lysozyme, antiprotease, and complement) and cellular (myeloperoxidase content, production of reactive oxygen and nitrogen species) responses and disease resistance against* Aeromonas hydrophila* were determined.* A. hydrophila* are Gram-negative straight rods with rounded ends (bacilli to coccobacilli shape) and are regarded as both fish and human pathogens. Lysozyme activity significantly increased after feeding fish with aqueous extract for 1, 2, or 3 weeks. Reactive oxygen species production and myeloperoxidase content showed significant enhancement after 1 week of feeding with aqueous extract. The percent mortality in fish following feeding the extract also diminished significantly when challenged with the pathogen [99].

The immunomodulatory responses of methanol extract of whole plant of* E. alba* (containing 1.6% wedelolactone) have been assessed at five dose levels (dose-response relationship) ranging from 100 to 500 mg/kg body wt. using carbon clearance, antibody titer, and cyclophosphamide immunosuppression parameters.* E. alba* extract significantly increased the phagocytic index and antibody titer. The *F* ratios of the phagocytic index and white blood cell (WBC) count were also significant [100].

### 4.12. Antiepilepsy Activity

Methanol extraction of leaf powder of* E. alba* was evaluated for its antiepileptic activity through Maximal Electroshock Test (MES) in rats. The extract was administered orally to rats for 7 days at doses of 50, 100, and 200 mg per kg body weight. One hour after the last treatment, seizures were induced in rats by delivering electroshock of 150 mA for 0.2 s with an electroconvulsiometer through a pair of ear clip electrodes. A decrease in duration of hind leg extension was taken as a parameter for anticonvulsant activity. Compared to controls, rats administered extract at different doses exhibited significant decrease in the duration of time spent in extensor phase in a dose-dependent manner. The antiepileptic activity was attributed to wedelolactone, luteolin, and *β*-amyrin present in the extract [101].

The anticonvulsant activity of methanol extract of* E. alba* leaves was studied using pentylenetetrazole- and picrotoxin-induced seizure models in mice and guinea pigs. The mechanism was further elucidated by studying the extract's GABA_A_ receptor modulatory activity and its effect on levels of GABA (*γ*-amino butyric acid) in mice brain. Potent anticonvulsant activity was demonstrated by the extract when administered at doses of 10–200 mg/kg with a saturation level at 50 mg/kg. The observed anticonvulsant effect was attributed to positive modulatory effects on GABA_A_ receptors [102]. Wedelolactone and luteolin, present in the extract, were hypothesized for giving the observed effect. Notably, GABA_A_ receptor dysfunction contributes to epileptogenesis [103]. Wedelolactone has been reported to have selectivity and affinity towards BZD (benzodiazepine) binding site on GABA_A_ receptors [104]. Also luteolin has neuroprotective activity and has affinity towards BZD binding site on the GABA_A_ receptors [105].

*E. alba* ethanolic leaf extracts at doses of 50, 100, 200, and 400 mg/kg, p.o., were studied for anticonvulsant and muscle relaxant activity on maximal electroshock-induced seizures (MES), rotarod, and traction test, respectively, in rats. At doses of 200 and 400 mg/kg, the extract reduced seizures induced by MES, decreased the duration of tonic hind limb extension (THLE) (by 76.2 and 89.8%, resp.), and decreased motor coordination showing anticonvulsant and muscle relaxant activity [106].

*E. alba* ethanolic leaf extract has been shown to cause thiopental sodium-induced sleeping time in rats at an early stage and to prolong the duration of sleep at doses of 200 and 400 mg/kg. The extract at 400 mg/kg also decreased locomotor activity in rats, thus showing a sedative effect. Ursolic and oleanolic acids, present in the extract, can act as GABA_A_ agonists and this property can be responsible for the central nervous system (CNS) depressant effect [107]. Notably, CNS depressant and antiepileptic activities have been reported for methanolic extract of leaves of* Ipomoea aquatica* [108].

### 4.13. Snake Bite

Extract of* E. alba* has been shown to inhibit snake venom phospholipase A2 activity of* Crotalus durissus terrificus* venom. The inhibitory activity has been attributed to the coumestans, wedelolactone, and demethylwedelolactone, present in the extract [109].

### 4.14. Anticancer Activity

The anticancer potential of hydroalcoholic extract of* E. alba* has been evaluated. The extract inhibited the cell proliferation in dose-dependent manner in HepG2, A498, and C6 glioma cell lines with an IC_50_ of 22 ± 2.9, 25 ± 3.6, and 50 ± 8.7 *μ*g/mL, respectively. The expression of matrix metalloproteinases (MMP) 2 and 9 was downregulated significantly. Additionally, downregulation of nuclear factor *κ*B (NF*κ*B) was also observed. DNA damage was observed following 72 h of extract treatment, leading to apoptosis [110]. Hydroalcoholic extract of the plant also demonstrated antiproliferative activities in multidrug-resistant DR-HepG2 cells, a hepatocellular carcinoma cell line [111].

Juice obtained from* E. alba* was shown to inhibit the migration of HCC-S102 (hepatocellular carcinoma) cells. In various human cancer cell lines of different tissue origins (liver, lung, and breast), the juice inhibited migration of all the cell lines with IC_50_ values ranging from 31–70 *μ*g/mL. Thus the plant has potential for preventing cancer metastasis [112]. Antiangiogenic activity was also demonstrated with the juice.

The ethyl acetate, methanol, and aqueous extracts of whole dried plants of* E. alba* were assessed for their inhibitory effects on the human lung epithelial adenocarcinoma cell line (HCC-827) using the MTT assay. Dose-dependent reductions in viable cell count were noticed with all three extracts with the ethyl acetate extract showing the most potency. All extracts induced apoptosis in the cancer cells [113].

Ethinylestradiol is widely used in various contraceptives and for treatment of metabolic and sexual disorders. It is also a genotoxic and a tumor initiating agent. The treatment of 10 *μ*M of ethinylestradiol along with 1.02 × 10^−4^, 2.125 × 10^−4^, 3.15 × 10^−4^, and 4.17 × 10^−4^ g/mL acetone extract of* E. alba* leaves resulted in a significant dose-dependent decrease in the genotoxic effects induced by the treatment of 10 *μ*M of ethinylestradiol in cultured human lymphocytes [114].

Crude methanol extract of* E. alba* has been shown to inhibit growth of colon cancer cells [115]. The methanolic extract of the aerial parts of the plant showed inhibitory activity on the proliferation of hepatic stellate cells or HSCs. Activity-guided fractionation led to the isolation of five oleanane-type triterpenoids, echinocystic acid, eclalbasaponin II, eclalbasaponin V, eclalbasaponin I, and eclalbasaponin III, which are all echinocystic acid derivatives. Among the five echinocystic acid derivatives isolated, echinocystic acid and eclalbasaponin II significantly inhibited the proliferation of HSCs in dose- and time-dependent manners [116].

### 4.15. Antiulcer Activity

The ethanolic extract of* E. alba* has been examined for its antiulcer effects in several ulcer models in rats, like cold resistant stress (CRS) and pylorus ligation (PL). The extract administered orally twice daily at doses of 50, 100, and 200 mg/kg was found to dose-dependently and significantly reduce ulcerative lesions. At the same time, extract administration led to significant attenuation of lipid peroxidation and elevated levels of catalase activity. Antisecretory activity of the extract was evidenced by significant reduction in gastric volume, acid output, and increase in gastric pH when compared to control (without extract) rats [117].

The methanolic extract of* E. alba* also showed antiulcer activity in ulcers induced in thirty- six-hour fasted Sprague Dawley rats by aspirin or ethanol or pylorus ligation plus aspirin treatment. In all the three separate experiments the group receiving oral administration of* E. alba* prior to ulcer induction showed highly significant reduction in the occurrence of gastric ulcers as well as gastric inflammation (after 4 h of treatment) as compared to the control groups. The extract activity was comparable to the activity of the proton pump inhibiting drug rabeprazole [118].

### 4.16. Anthelmintic Activity

The methanol extract of whole plant of* E. alba* was evaluated for its anthelmintic potential against the earthworm* Pheretima posthuma* at doses of 25–100 mg/mL. The extract exhibited paralysis of worms at doses of 50, 75, and 100 mg/mL and caused death of worms at 75 and 100 mg/mL [119]. The ethanol and aqueous extract also showed anthelmintic activity against* P. posthuma* [120].

## 5. Pharmacological Activity Reports on* E. alba* Phytoconstituents

### 5.1. Wedelolactone

*In vitro* 5-lipoxygenase inhibition by wedelolactone has been reported [121]. 5-Lipoxygenase (5-LO) catalyzes the two-step conversion of arachidonic acid to leukotriene A4 (LTA4) [122].

A study with synthetically prepared wedelolactone and derivatives showed that both the parent compound and most of the wedelolactone derivatives significantly protected primary cultured liver cells from the toxicity of CCl_4_, galactosamine (Galc), and phalloidin and strongly inhibited the activity of 5-lipoxygenase in porcine leukocytes. The synthetic wedelolactone was also found to have stimulatory effect on the RNA synthesis in isolated nuclei from hepatocytes [123].

Ethanolic extract of the aerial parts of* E. alba* has been shown to neutralize the lethal activity of the venom of South American rattlesnake (*Crotalus durissus terrificus*) when mixed* in *vitro before i.p. injection into adult Swiss mice. Three phytoconstituents isolated from the plant, namely, wedelolactone (0.54 mg/animal), sitosterol (2.3 mg/animal), and stigmasterol (2.3 mg/animal), were able to neutralize three lethal doses of the venom. Aqueous extracts of the plant inhibited the release of creatine kinase from isolated rat muscle exposed to the crude venom. This protection was also observed* in vivo*, when the venom was preincubated with the extract prior to injection into mice [124].

The antimyotoxic and antihemorrhagic effects of* E. alba* and three of its constituents, wedelolactone, sitosterol, and stigmasterol, have been investigated for their ability to protect against myotoxicity of crotalid venoms (*Bothrops jararaca, Bothrops jararacussu*, and* Lachesis muta*) and purified myotoxins (bothropstoxin, BthTX; bothropasin; and crotoxin) through quantification* in vitro* by the release rate of creatine kinase (CK) from rat or mouse extensor digitorum muscles and* in vivo* by the plasma CK activity in mice. Wedelolactone was more effective than sitosterol or stigmasterol to neutralize* in vitro* myotoxicity of the crotalid venoms and myotoxins. The* in vivo* myotoxicity of venoms and myotoxins was also neutralized by preincubation with wedelolactone. Intravenous administration of wedelolactone attenuated the increase in plasma CK activity induced by subsequent intramuscular injections of the crotalid venoms or the myotoxins. Wedelolactone inhibited the hemorrhagic effect of* B. jararaca* venom, as well as the phospholipase A2 activity of crotoxin and the proteolytic activity of* B. jararaca* venom. These effects have been attributed to antiproteolytic and antiphospholipase A2 activities of the constituents [125]. Wedelolactone also antagonized the myotoxic activity in mice of venoms from* Crotalus viridis viridis* and* Agkistrodon contortrix laticinctus* and two phospholipase A2 myotoxins, CVV myotoxin and ACL myotoxin, isolated from them [126].

Wedelolactone has been found to inhibit lipopolysaccharide- (LPS-) induced caspase-11 (an inflammatory caspase) expression in cultured cells by inhibiting NF-*κ*B-mediated transcription. It has further been shown that wedelolactone is an inhibitor of IKK (I*κ*B, part of the upstream NF*κ*B signal transduction cascade), a kinase critical for activation of NF-*κ*B by mediating phosphorylation and degradation of I*κ*B*α* [127]. Wedelolactone also reportedly significantly inhibited the protein expression levels of iNOS (inducible nitric oxide synthase, produced after activation by endotoxins or cytokines and generating copious amounts of NO) and COX-2 (cyclooxygenase-2 converts arachidonic acid to prostaglandins, resulting in pain and inflammation) in LPS-stimulated RAW 264.7 cells, as well as the downstream products, including NO (nitric oxide), PGE2 (prostaglandin E2), and TNF-*α* (tumor necrosis factor-*α*). Moreover, wedelolactone also inhibited LPS-induced NF-*κ*B p65 activation via the degradation and phosphorylation of I*κ*B-*α* and subsequent translocation of the NF-*κ*B p65 subunit to the nucleus [128]. This suggests that the compound can be used as an anti-inflammatory agent.

Bioassay-guided fractionation for anti-HIV-1 (human immunodeficiency virus 1) integrase activity led to isolation of six compounds from* E. alba* extract. They were identified as 5-hydroxymethyl-(2,2′:5′,2′′)-terthienyl tiglate, 5-hydroxymethyl-(2,2′:5′,2′′)-terthienyl agelate, 5-hydroxymethyl-(2,2′:5′,2′′)-terthienyl acetate, ecliptal, orobol, and wedelolactone. Wedelolactone showed maximum anti-HIV-1 integrase inhibitory activity with an IC_50_ value of 4.0 ± 0.2 microns. This study supports the use of* E. alba* in acquired immunodeficiency syndrome (AIDS) patients [129].

Wedelolactone, isolated from* Wedelia chinensis*, has been found to modulate the androgen receptor (AR) activation of transcription from prostate-specific antigen promoter in prostate cancer (PCa) cells [130]. The anticancer activity of wedelolactone has also been shown in androgen receptor-negative MDA-MB-231 breast cancer cells, where wedelolactone suppressed growth and induced apoptosis. Cells were arrested at the S and G2/M phase of the cell cycle with induction of DNA damage. Wedelolactone was found to interact with dsDNA and inhibited the activity of DNA topoisomerase II*α* [131].

In* Chlamydia trachomatis*-infected HEp-2 cells (human epithelial type 2 cells, considered to originate from a human laryngeal carcinoma), wedelolactone was found to induce apoptosis in combination with LPS and polyI:C (polyinosinic:polycytidylic acid) leading to lesser viability of* Chlamydia* [132].

G protein-coupled receptor-35 (GPR35) has been shown to be a target of the asthma drugs cromolyn disodium and nedocromil sodium. Wedelolactone, which is antiallergic, was found to be a potent *β*-arrestin-biased GPR35 agonist and can be considered a potential drug against asthma [133].

Adipocyte hyperplasia is associated with obesity and arises due to adipogenic differentiation of resident multipotent stem cells in the vascular stroma of adipose tissue and remote stem cells of other organs [134]. Wedelolactone has been observed to inhibit the adipogenic differentiation of human adipose tissue-derived mesenchymal stem cells (hAMSCs). It has been shown that this process may be mediated through the ERK (extracellular signal regulated kinase) pathway [135].

Metabolism of arachidonic acid through the 5-lipoxygenase pathway has been shown to play a critical role in the survival of prostate cancer cells. Wedelolactone has been found to kill both androgen-sensitive and androgen-independent prostate cancer cells in a dose-dependent manner by inducing apoptosis. Wedelolactone-induced apoptosis was dependent on c-Jun N-terminal kinase (c-JNK) and caspase-3. Apoptosis was triggered via downregulation of protein kinase C*ε* but without inhibition of Akt (protein kinase B), suggesting that a novel mechanism is at work [136]. Notably, JNK plays a critical role in death receptor-initiated extrinsic as well as mitochondrial intrinsic apoptotic pathways through modulating the activities of mitochondrial pro- and antiapoptotic proteins through distinct phosphorylation events [137]. Caspase-3 is a death protease and is frequently involved in apoptosis [138]. Downregulation of protein kinase C*ε* has also been shown to occur using tumor necrosis factor-related apoptosis inducing ligand (TRAIL) stimulated glioma cells; however, in this case reduced expression of Akt was also observed [139].

Wedelolactone has been reported to synergize with interferon-*γ* (IFN-*γ*) to induce apoptosis in tumor cells. The compound increased IFN-*γ* signaling by inhibiting STAT1 (signal transducer and activator of transcription 1 protein) dephosphorylation and prolonging STAT1 activation through specific inhibition of T-cell protein tyrosine phosphatase (TCPTP), an important tyrosine phosphatase for STAT1 dephosphorylation [140]. STAT1 has been implicated in modulating pro- and antiapoptotic genes following stress-induced responses [141].

Wedelolactone has been reported to exhibit antifibrotic effects on human hepatic stellate cell line LX-2. The compound reduced the cellular viability of LX-2 in a time- and dose-dependent manner. Wedelolactone induced apoptosis of LX-2 cells by decreasing the expression of antiapoptotic Bcl-2 and increasing the expression of proapoptotic Bax. The inhibition of activation of LX-2 cells has been demonstrated and attributed by the authors to a number of reasons including inducing Bcl-2 family involved apoptosis, upregulating phosphorylated status of ERK and JNK expressions, and inhibiting nuclear factor-*κ*B (NF-*κ*B) mediated activity [142].

Overall, the various reports indicate that the compound has antiinflammatory, hepatoprotective, snake venom neutralizing, anti-HIV, anticancer, and antiasthmatic effects.

### 5.2. Eclalbasaponins

The antiproliferative effect of eclalbasaponinsisolated from* E. alba* on hepatic stellate cells has previously been described [116]. Eclalbasaponin I isolated from aerial parts of the plant reportedly dose-dependently inhibited the proliferation of hepatoma cell smmc-7721 with IC_50_ value of 111.1703 *μ*g/mL [143]. Eclalbasaponin VI, isolated from* E. alba*, has been shown to demonstrate *α*-glucosidase activity [64]. Eclalbasaponin, isolated from* E. alba*, has been shown to demonstrate antibacterial activity [92].

Echinocystic acid isolated from ethyl acetate fraction of 70% ethanol extract of the plant inhibited LPS-induced production of nitric oxide and cytokines such as tumor necrosis factor-*α* and interleukin-6 in RAW 264.7 macrophages. The compound also inhibited LPS-induced inducible nitric oxide synthase expression at the protein level and inducible nitric oxide synthase (iNOS), tumor necrosis factor-*α* (TNF-*α*), and interleukin-6 (IL-6) expression at the mRNA level and inhibited LPS-induced iNOS promoter binding activity. Additionally, echinocystic acid suppressed the lipopolysaccharide-induced transcriptional activity of nuclear factor-*κ*B by blocking the nuclear translocation of p65 [144]. Thus this compound can be regarded as a potent anti-inflammatory agent.

Taken together, eclalbasaponins possess anticancer and anti-inflammatory effects.

### 5.3. *α*-Amyrin

Reports on *α*-amyrin are few; however, several reports are present on pharmacological effects of a combination of *α*- and *β*-amyrin (ABA) as well as *α*-amyrin derivatives. Antilipoxygenase activity has been reported for *α*-amyrin acetate, suggesting its possibly beneficial role in arthritis [145]. In adult male Wistar rats made arthritic by subplantar injection of complete Freund's adjuvant, *α*-amyrin palmitate caused increases in serum hyaluronate and blood granulocytes toward nonarthritic levels and corrected the moderate anemia of adjuvant arthritis [146]. The triterpenes, *α*-amyrin and its palmitate and linoleate esters caused growth inhibition of rat osteosarcoma cells and tadpole collagenase digestion of type I (bone) native collagen. The antiarthritic effect has been attributed to inhibition by the triterpenes of joint destruction [147].

ABA, isolated from the resin of* Protium kleinii*, caused dose-dependent and significant antinociception against the visceral pain in mice produced by (intraperitoneal) i.p. injection of acetic acid; i.p., p.o., intracerebroventricular (i.c.v.), or intrathecal (i.t.) administration of ABA inhibited both neurogenic and inflammatory phases of the overt nociception caused by intraplantar (i.pl.) injection of formalin; ABA given by i.p., p.o., i.t., or i.c.v. routes inhibits the neurogenic nociception induced by capsaicin. In addition, i.p. treatment with ABA was able to reduce the nociception produced by 8-bromo-cAMP (8-Br-cAMP) and by 12-O-tetradecanoylphorbol-13-acetate (TPA) or the hyperalgesia caused by glutamate [148].

Anti-inflammatory effect of *α*-amyrin isolated from* P. kleinii* has been demonstrated through its ability to inhibit both ear edema and influx of polymorphonuclear cells in response to topical application of 12-O-tetradecanoylphorbol-acetate (TPA) in mice ears [149]. In TPA-induced skin inflammation in mice, topical application of *α*-amyrin inhibited TPA-induced increase of prostaglandin E2 (PGE2) levels. *α*-Amyrin also prevented I*κ*B*α* degradation, p65/RelA phosphorylation, and NF-*κ*B activation. In addition, *α*-amyrin also inhibited the activation of upstream protein kinases, namely, ERK, p38 mitogen-activated protein kinase (MAPK), and protein kinase C (PKC) *α*. The anti-inflammatory mechanism has been proposed to be suppression of COX-2 expression through inhibition of ERK, p38MAPK, and PKC*α*, as well as blocking NF-*κ*B activation [150].

ABA, isolated from* Protium heptaphyllum*, significantly attenuated cerulein-induced acute pancreatitis in mice. Decreases in cerulein-induced increases of tumor necrosis factor TNF-*α*, interleukin-6, lipase, amylase, myeloperoxidase (MPO), and TBARS were noted with administration of ABA. Moreover, ABA significantly suppressed the pancreatic edema, inflammatory cell infiltration, acinar cell necrosis, and expressions of TNF*α* and iNOS [151]. Similar protective effect of ABA was also seen in acute pancreatitis induced in rats with L-arginine [152].

ABA (3–100 mg/kg), isolated from* Protium heptaphyllum* resin, demonstrated significant antinociceptive activity against either subplantar (1.6 *μ*g) or intracolonic application of capsaicin in mice [153]; antinociceptive activity of ABA has also been reported in mice for cyclophosphamide-induced bladder pain and intracolonic administration of mustard oil [154].

The protective effect of ABA has been demonstrated against trinitrobenzene sulphonic acid- (TNBS-) induced colitis in Swiss male mice [155]. The results indicated that ABA suppresses inflammatory cytokines and COX-2 levels possibly via inhibition of NF-*κ*B and CREB-signaling pathways. The preventive effect of ABA against dextran sulfate-induced colitis in mice has also been demonstrated; it has been hypothesized that the cannabinoid pathway may be involved [156].

*α*-Amyrin acetate, isolated from* Tylophora hirsuta*, tested positive for antispasmodic activity on spontaneous rabbits' jejunum preparations with EC_50_ value of (60 ± 2) × 10(−5) M. The compound also tested positive on KCl induced contractions with EC_50_ value of (72 ± 3) × 10(−5) M. The compound thus can prove to be of use against gastrointestinal disorders like diarrhea and dysentery [157].

ABA, isolated from the resin of* Protium heptaphyllum*, manifested a hypolipidemic effect in normoglycemic mice and reduced the elevated plasma glucose levels during oral glucose tolerance tests. STZ-induced diabetic mice showed significant decreases in blood glucose (BG), total cholesterol (TC), and serum triglycerides (TGs), when treated with ABA. Histopathological studies showed the beneficial effect of ABA on pancreas in maintaining *β*-cell integrity. In high fat diet- (HFD-) fed mice oral administration of ABA (10, 30, and 100 mg/kg), the HFD-associated rise in serum TC and TGs was significantly less, particularly at a dose of 100 mg/kg. At this dose, there were significant decreases in very low-density lipoprotein (VLDL) and low- density lipoprotein (LDL) cholesterol and an elevation of high density lipoprotein (HDL) cholesterol. The atherogenic index was also significantly reduced by ABA [158].

*α*-Amyrin acetate, isolated from aerial roots of* Ficus benghalensis*, has been shown to reduce hyperglycemia and improve diabetic conditions in STZ-induced diabetic rats and db/db diabetic mice [159].

*α*-Amyrin, isolated from* Rhaponticum carthamoides*, has been shown to induce proliferation of human keratinocytes (HaCaT) by about 18% [160].

*α*-Amyrin acetate at concentrations of 1.6% caused 76.9% mortality rate in adult female* A. stephensi* mosquitoes.* In vivo* exposure of the mosquitoes to the compound was found to increase mean probing time and decrease blood engorgement time and feeding rate and a declination in fecundity, which reduced the overall survival and reproductive capacity of the malaria vector* A. stephensi* [161].

ABA, from the stem bark resin of* Protium heptaphyllum*, showed anxiolytic and antidepressant effects when tested in mice by the open-field, elevated plus maze, rotarod, forced swimming, and pentobarbital-induced sleeping time. It has been hypothesized that the anxiolytic effect may involve benzodiazepine-type receptors, while the antidepressant effect may involve noradrenergic mechanisms [162].

Hepatoprotective action of ABA has been reported against acetaminophen-induced liver injury in mice. ABA was isolated from the trunk wood resin of* Protium heptaphyllum*. Acetaminophen (500 mg/kg, p.o.) caused fulminant liver damage characterized by centrilobular necrosis with inflammatory cell infiltration, an increase in serum ALT and AST activities, a decrease in hepatic glutathione (GSH) and 50% mortality. Pretreatment with ABA (50 and 100 mg/kg, i.p. at 48, 24, and 2 h before acetaminophen) attenuated the acetaminophen-induced acute increase in serum ALT and AST activities, increased the depleted hepatic GSH, and considerably reduced the histopathological alterations. Furthermore, ABA potentiated the pentobarbital (50 mg/kg, i.p.) sleeping time. The results suggest that the hepatoprotective action of ABA involved possible suppression of liver cytochrome P450 and diminution in oxidative stress and toxic metabolite formation in liver [163].

ABA, isolated from* Protium heptaphyllum* resin, showed gastroprotective effect against ethanol-induced gastric mucosal damage in mice. Maximal gastroprotection was observed with a 100 mg/kg dose of ABA, which was almost abolished in mice with their sensory afferents chemically ablated by a neurotoxic dose of capsaicin. It has been suggested that the gastroprotective mechanism of ABA involves at least in part the activation of capsaicin-sensitive primary afferent neurons [164].

ABA, isolated from* Protium heptaphyllum* resin, reportedly significantly inhibited the scratching behavior induced by dextran T40 and compound 48/80 in mice. The inhibition has been attributed to a stabilizing action on mast cell membrane [165].

The available scientific literature suggests that *α*-amyrin and derivatives by themselves or a combination of *α* and *β*-amyrins have diverse pharmacological activities including antiarthritic, analgesic, anti-inflammatory, antispasmodic, antidiabetic, cholesterolemic, antimalarial, anxiolytic, antidepressant, hepatoprotective, and gastroprotective activities and also may be beneficial against pancreatitis and pruritus.

### 5.4. Miscellaneous Phytochemical Constituents of* E. alba*

Several phytochemical constituents of* E. alba* have reported multiple and diverse pharmacological activities, which will only be briefly discussed. Oleanolic acid is known for both antidiabetic and anticancer effects. It can directly modulate enzymes connected with insulin biosynthesis, secretion, and signaling [166]. Many of its effects are mediated through activation of the transcription factor Nrf2 (nuclear factor erythroid 2-related factor 2). Nrf2 can modulate the expression of more than 200 genes that are crucial in the metabolism of drugs and toxins, protection against oxidative stress and inflammation, and maintaining protein stability and degradation. Nrf2 can interact with tumor suppressor protein 53 (p53) and so control cell cycle and NF-*κ*B. Through these interactions, Nrf2 plays a major role in protecting against many age-related diseases including cancer and neurodegeneration, as well as increasing longevity [167].

In tumor cells, a recent review has pointed out that the compound can modulate multiple signaling pathways, like NF-*κ*b, AKT, signal transducer and activator of transcription 3, mammalian target of rapamycin, caspases, intercellular adhesion molecule 1, vascular endothelial growth factor, and poly (ADP-ribose) polymerase [168]. As such, oleanolic acid can be a potential preventive as well as a therapeutic agent for cancer.

Ursolic acid has been shown in various reports to have antioxidative, anticancer, and anti-inflammatory properties, although a recent review has pointed out that proinflammatory properties of the compound have also been reported in normal cells and tissues [169]. A number of reports have shown ursolic acid to have anticancer, cytotoxic, antitumor, antioxidant, anti-inflammatory, antiwrinkle, anti-HIV, acetyl cholinesterase, *α*-glucosidase, antimicrobial, and hepatoprotective activities (reviewed in [170]).

Oleanolic and ursolic acids have reportedly hepatoprotective, anti-inflammatory, and antihyperlipidemic properties [171]. Oleanolic and ursolic acids can also be potentially useful in neurodegenerative disorders like Alzheimer's disease [172]. A review study has described the anticancer effects of ursolic acid to include protection of cellular DNA from damage, inhibition of epidermal growth factor receptor/mitogen-activated protein kinase signal or of FoxM1 transcription factors, antiangiogenesis (which can inhibit tumor cell growth), inhibition of tumor cell migration and invasion, and inducing apoptosis in cancer cells [173]. The forkhead transcription factor or FoxM1 has been shown to bind and regulate a group of genes which are mainly involved in controlling late cell cycle events in the G2 and M phases [174]. Inhibition of FoxM1 expression has been found to diminish the proliferation and anchorage-independent growth of breast cancer cells [175].

Antioxidant, anti-inflammatory, and antiallergic activities have been described for luteolin. The compound may also have cardioprotective effect [176]. Luteolin has also been described to have the ability to block the development of cancer cells both* in vitro* and* in vivo* through offering protection from carcinogenic stimuli, inhibiting tumor cell proliferation, and induction of cell cycle arrest and/or apoptosis [177]. Furthermore, luteolin can sensitize cancer cells to therapeutic-induced cytotoxicity through a variety of mechanisms including suppression of cell cycle pathways like phosphatidylinositol 3′-kinase (PI3K)/Akt, NF-*κ*B, and X-linked inhibitor of apoptosis protein (XIAP) and stimulating apoptosis pathways including those that induce the tumor suppressor p53 [178]. Modulation of reactive oxygen species (ROS) levels, inhibition of topoisomerases I and II, reduction of NF-*κ*B and AP-1 activity, stabilization of p53, and inhibition of PI3K, STAT3, insulin-like growth factor 1 receptor (IGF1R), and human epidermal growth factor receptor 2 (HER2) have also been described as the causes behind the cancer chemopreventive and chemotherapeutic potential of luteolin [179]. It is to be noted that overexpression of HER2 is associated with certain aggressive forms of breast cancer.

The cardioprotective role of luteolin has been shown in cardiomyocytes following ischemia-reperfusion, suggesting that the compound can form the basis for preventing and treating cardiovascular diseases [180].

Apigenin has been described as a chemopreventive agent [181]. The compound also has antioxidant and anti-inflammatory properties. The compound may also have a beneficial effect in cardiovascular and neurological disorders [182]. A recent review has pointed out that apigenin can inhibit cancer cell growth, sensitize cancer cells to elimination by apoptosis, and hinder the development of blood vessels to serve the growing tumor. Apigenin is able to reduce cancer cell glucose uptake, inhibit remodeling of the extracellular matrix, inhibit cell adhesion molecules that participate in cancer progression (like VCAM-1 [183]), and oppose chemokine signaling pathways that direct the course of metastasis into other locations. For instance, apigenin has been shown to suppress migration and invasion of transformed cells through downregulation of C-X-C chemokine receptor 4 expression [184]. All of these effects have the net result of blocking progression and metastasis of cancer [185]. In head and neck cancers, apigenin may act through inhibiting GLUT-1 expression [186].

## 6. Conclusion

The plant,* E. alba*, is regarded by traditional medicinal practitioners as a valuable medicinal plant, particularly for the treatment of liver disorders, gastrointestinal disorders, respiratory tract disorders, hair loss, skin disorders, and fever. Scientific evidences have validated most of the claims of the ethnomedicinal uses, including that of treatment of snake bite with the plant. Various important phytochemicals have been isolated and identified from the plant. These compounds include wedelolactone, eclalbasaponins, *α*-amyrin, ursolic acid, oleanolic acid, luteolin, and apigenin. The available scientific reports indicate that these compounds can form the next generation of drugs to treat cancer, arthritis, liver diseases, hair loss, and snake bites.

## Figures and Tables

**Table 1:** Taxonomic hierarchy of *E. alba*.

Kingdom	Plantae	
	Subkingdom	Viridaeplantae
	Infrakingdom	Streptophyta
	Division	Tracheophyta
	Subdivision	Spermatophytina
	Infradivision	Angiospermae
	Class	Magnoliopsida
	Superorder	Asteranae
	Order	Asterales
	Family	Asteraceae
	Genus	Eclipta L.
	Species	Eclipta alba (L.) Hassk.

**Table 2:** Reported phytoconstituents of *E. alba* [2, 6–8].

Nature of phytoconstituent(s)	Phytoconstituent(s)
Coumestan	Wedelolactone, demethylwedelolactone, demethylwedelolactone-7-glucoside

Terpenoids and their glycosides	Eclalbasaponins VII–X (taraxastane triterpene glycosides), eclalbasaponins I–VI (oleanane triterpene glycosides), eclalbosaponins I–VI (triterpene glycosides), ecliptasaponins C and D (triterpenoid glucosides), *α*-amyrin, oleanolic acid, ursolic acid (triterpenoids)

Sterol	Stigmasterol, daucosterol, stigmasterol-3-*O*-glucoside

Alkaloids	[(20*S*)(25*S*)-22,26-imino-cholesta-5,22(*N*)-dien-3*β*-ol] (verazine), [20-*epi*-3-dehydroxy-3-oxo-5,6-dihydro-4,5-dehydroverazine], [(20*R*)-20-pyridyl-cholesta-5-ene-3*β*,23-diol] (ecliptalbine), [(20*R*)-4*β*-hydroxyverazine], [4*β*-hydroxyverazine], [(20*R*)-25*β*-hydroxyverazine], [25*β*-hydroxyverazine]

Flavonoids	Luteolin-7-glucoside, luteolin, apigenin, orobol (isoluteolin)

Sesquiterpene lactones	5-hydroxymethyl-(2,2′:5′,2′′)-terthienyl tiglate, 5-hydroxymethyl-(2,2′:5′,2′′)-terthienyl agelate, 5-hydroxymethyl-(2,2′:5′,2′′)-terthienyl acetate

Terthienyl aldehyde	Ecliptal

Fatty alcohols	Hentriacontanol, heptacosanol

Volatile oils	Heptadecane, 6,10,14-trimethyl-2-pentadecanone, *n*-hexadecanoic acid, pentadecane, eudesma-4(14),11-diene, phytol, octadec-9-enoic ecid, 1,2-benzenediacarboxylic acid diisooctyl ester, (*Z*,*Z*)-9,12-octadecadienoic acid, (*Z*)-7,11-dimethyl-3-methylene-1,6,10-dodecatriene, (*Z*,*Z*,*Z*)-1,5,9,9-tetramethyl-1,4,7-cycloundecatriene

Saponins	Eclalbatin (triterpene saponin), dasyscyphin C

Polyacetylinic compounds	*α*-Terthienylmethanol, polyacetylenes, polyacetylene substituted thiophenes

Phenolic acids	Protocatechuic acid, 4-hydroxy benzoic acid

**Table 3:** Reported ethnomedicinal uses of *E. alba*.

Location and tribe/nature of user(s)	Plant part(s) used, diseases treated, formulations
Traditional practitioners of Aligarh (1), Budaun (2), Bulandshahar (3), Farrukhabad (4), Hathras (5) districts of Western Uttar Pradesh, India [9] [Reported uses in the various districts are shown in the right column in parentheses]	Acidity. Plant decoction is administered thrice daily with cow milk before each meal for 15 days (1–5). Alopecia. Leaf extract is given orally twice a day for 3 months (1–5). Asthma. Whole plant ash is given orally thrice daily for 3 months (1–5). Body pain. Fresh leaf extract is given orally thrice daily for 5 days or till cure (1–5). Bronchitis and pneumonia. Whole plant decoction is given orally with honey twice a day for 7 days or till cure (1–5). Burns. Whole plant extract is given orally twice a day for 7 days. Leaf paste is applied externally. This is continued till cure (1–5). Constipation. Root powder is given orally once a day for 3 days (1–5). Diarrhea and dysentery. Whole plant decoction is given orally thrice daily for 7 days or till cure (1–5). Edema. Plant extract is given twice a day for 7 days or till cure. Fever. Whole plant extract is given orally twice or thrice daily for 7 days or till cure (1–5). General weakness. Whole plant extract mixed with 3 g fruit powder of *Phyllanthus emblica* is given orally twice a day for 6 weeks or till the person recovers from weakness (1–5). Gingivitis. Leaf extract is given orally twice a day for 3 weeks or till cure (1–5). Hemorrhoids. Root extract is administered orally thrice daily (1, 2). Hair fall. Leaf extract is given orally twice daily with cow milk for 3 months (1–5). High blood pressure. Plant decoction is given orally twice or thrice a day for 3 months or till the patient recovers fully (1–5). Jaundice. Fresh plant extract is given orally twice or thrice daily for 3 weeks or till cure. Leaf extract along with honey is given orally twice or thrice daily for 15 days or till cure. Plant extract mixed with plant extract of *Boerhavia diffusa* is given orally twice a day for 15 days or till cure (1, 2). Liver enlargement. Plant extract is given orally twice or thrice daily for 1 month or till cure. This therapy is administered to adult patients only (1–5). Loss of appetite. Leaf decoction is given orally before each meal twice daily for 15 days. Leaf powder is given orally after each meal for 15 days (1, 2, 6). Palpitation of heart. Leaf extract mixed with honey is given orally twice a day for 7 days or till cure (1–5). Paronychia or whitlo. Whole plant paste is applied externally (1–5). Pimples. Fresh leaf extract is given orally twice daily with cow milk for 2 months (1–5). Premature graying of hair. Fresh leaf extract is gently applied to hair (1–5). Skin diseases. Plant paste is applied externally for 15 days in eczema. Leaf paste is applied externally to boils and extract is given orally twice daily for 15 days (1, 2, 5). Spleen enlargement. Leaf extract mixed with honey is given orally twice or thrice daily for 15 days or till cure. Urinary tract infections. Plant extract is given orally twice a day for 15 days. The extract is also used to wash genitalia externally till cure (1–5). Weakness of vision. Leaf extract is given orally twice a day with cow milk for 3 months (1–3). Wounds. Leaf extract is used to wash open wounds (1, 3, 5). Wrinkles. Leaf extract with *Withania somnifera* root powder is given orally with cow milk twice daily for 3 months (1–5).

Local practitioners of Mount Abu in Rajasthan, India [10]	Leaves and flowers used for treatment of urinary problems, jaundice, asthma, and coughs.

Local community of Jalalpur Jattan, Gujrat district, Punjab, Pakistan [11]	Leaf paste applied to treat allergy, athelete's foot and ringworm.

Santal tribe residing in Thakurgaon district, Bangladesh [12]	Diabetes. Leaves of white-flowered plant are mixed with leaves of *Scoparia dulcis*, leaves of *Cynodon dactylon* and water and then boiled in an earthen vessel. The water is then strained through cloth and given to diabetic patients to be taken orally in the morning and evening on an empty stomach.

Inhabitants of Mansoora, Malegaon, India [13]	Plant is used as tonic, deobstruent, emetic and considered useful in enlargement of liver and spleen.

Chakma tribe of Tripura State, India [14]	Two teaspoons of leaf juice is administered daily against hepatic disorders.

Local people and tribal communities of Hanumangarh District, Rajasthan, India [15]	Plant is used as hair tonic. Extracted oil is used as tonic. Leaf juice is taken orally with honey in jaundice and dysentery. The plant is considered to stimulate the digestive system, augment appetite, and improve digestion. Leaf extract is given orally with water for diarrhea. The root is considered purgative and used in conditions of liver, spleen, and dropsy.

Inhabitants of Thar Desert, India [16]	Whole plant is considered deobstruent, antihepatotoxic, anticatarrhal, and febrifuge. Used in hepatitis, spleen enlargements, and skin diseases. Leaf is used to promote hair growth. Leaf extract in oil is applied to scalp before bedtime for insomnia.

Local communities and ethnic groups of Bundelkhand, Uttar Pradesh, India [17]	Decoction of plant used to treat scorpion sting.

Local herbalists of Samba District of Jammu and Kashmir State, India [18]	Whole plant is used in asthma, bronchitis, fever, gastric and hepatic disorders, jaundice, ulcers, wounds, sores, and leucoderma.

Folk medicinal practitioners of Rampal, Bagerhat District, Bangladesh [19]	Whole plant used to treat indigestion.

Tribals of Buldhana District, Maharashtra, India [20]	Whole plants and leaves used to treat wounds.

Malayali tribals of Kolli Hills, Eastern Ghats, Tamil Nadu, India [21]	Whole plant juice is given orally to treat snake bite.

Local people of Javadhu Hills, Tamil Nadu, India [22]	Plant is used for treatment of hepatitis.

Malayaraya tribes of Vannapuram village, Idukki, Kerala, India [23]	Whole plant is used for rejuvenating hair, kidneys, and liver.

Women of Kaibarta community of Assam, India [24]	Shoot juice with few drops of mustard oil or root extracts are given once daily for 3-4 days for diarrhea.

Local people of Mandi Bahauddin District, Pakistan [25]	Leaf paste applied for allergy, athlete's foot and ringworm.

Anyi-Ndenye pregnant women of Eastern Cote d'Ivoire, Africa [26]	Whole plant used to ensure fetal development and facilitate childbirth.

Local people of Dibrugarh, Assam, India [27]	Whole plant used as tonic and for treatment of spleen enlargement.

Women of Azamgarh District, Uttar Pradesh, India [28]	Whole plant juice with sugar is given to persons suffering from severe whitish dysentery.

Villagers of Nizamabad District, Andhra Pradesh, India [29]	Dry plant powder is given to elderly people to provide energy. Plant paste is applied to head to blacken gray hair.

Traditional herbal medical practitioners of Nagapattinam District, Tamil Nadu, India [30]	Leaf extract is applied on swellings.

Local people of Birbhum District in West Bengal, India [31]	Fresh leaves are applied with sesame oil to cure baldness, elephantiasis, and headache. Juice of whole plant is applied in skin disorders on affected areas of skin.

Saperas community of Khetawas, Jhajjar District, Haryana, India [32]	Treatment of snake bite.

Local traditional healers of Western Uttar Pradesh, India [33]	Decoction of whole plant is given for scorpion sting.

Bhil, Pawara and Pardhi tribes in Satpuda Mountain of Nandurbar, Dhule and Jalgaon district of Maharashtra, India [34]	4-5 powdered leaves are administered with a cup of water in a single dose for 2 days for menorrhagia.

Uraly tribes of Idukki District, Kerala, India [35]	Crushed leaves are applied on cuts and wounds.

Local inhabitants of rural and remote areas of Kalyanpur block of Kanpur District, Uttar Pradesh, India [36]	2–5 g leaf paste is applied on fresh cuts and wounds.

Gujjar tribes in the Shivalik Hills of Haridwar, Uttarakhand, India [37]	Jaundice, premature graying and falling of hair.

Tribes of Parambikulam Wildlife Sanctuary, Kerala, India [38]	Leaf paste is applied to hair to promote growth.

Local people and traditional healers of Ambala District, Haryana, India [39]	Leaf decoction is put on head to cure headache. Leaf extract is given to cure asthma, cold and for hair cleaning and lice.

Traditional healers and local people of Arghakhanchi District, Nepal [40]	Plant juice is applied externally in cuts and wounds.

Ethnic communities of Moradabad District, Western Uttar Pradesh, India [41]	Leaf extract is applied to head to get rid of dandruff and to blacken gray hair.

Tribals of Boudh District, Odisha, India [42]	Whole plant is grounded with black pepper and made into small pills. Two pills are administered twice a day to infants for treatment of jaundice and fever.
